# Atheroembolic Blue Toe Syndrome Progressing to Below-Knee Amputation Despite Attempted Source Control and Revascularization: A Case Report

**DOI:** 10.7759/cureus.117402

**Published:** 2026-10-03

**Authors:** David D Echavarria, Itamar Gal, Santiago T Espinoza, Aiden Gershbein, Daryl Gershbein

**Affiliations:** 1 College of Arts and Sciences, Florida State University, Tallahassee, USA; 2 Department of Urology, National Medical Association and Research Group, Gainesville, USA; 3 Department of Nursing, Florida State University, Tallahassee, USA; 4 Shepard Broad College of Law, Nova Southeastern University, Davie, USA; 5 Department of Podiatry, Miami Foot Center, Miami, USA

**Keywords:** atheroembolism, blue toe syndrome, chronic limb-threatening ischemia, distal ischemia, limb salvage

## Abstract

Blue toe syndrome is characterized by painful blue or violaceous discoloration of one or more toes caused by impaired digital arterial perfusion. We present a 72-year-old woman with peripheral arterial disease, diabetes mellitus, antiphospholipid syndrome, and prior thromboembolic disease who developed dusky-blue discoloration of the left third toe. Initial arterial duplex demonstrated no major arterial occlusion, while computed tomography angiography with runoff showed distal infrarenal aortic soft plaque and distal tibial/pedal arterial disease. The aortic lesion was considered the suspected proximal embolic source in the setting of concomitant distal tibial and pedal arterial disease.

The patient underwent endovascular aortic repair for proximal source control, but the left third toe nevertheless progressed to dry gangrene and required amputation. Over the following months, she developed nonhealing wounds, infections with *Pseudomonas aeruginosa* and methicillin-resistant *Staphylococcus aureus* (MRSA), worsened distal perfusion, and osteomyelitis. There were multiple limb salvage procedures, including angioplasty, toe amputation, common plantar embolectomy, plantar bypass, transmetatarsal amputation, Achilles tendon lengthening, antibiotic bead placement, and external fixation. Following these procedures, however, the foot developed worsening ischemic and infected tissue loss and needed an emergency left below-knee amputation.

Final pathology demonstrated significant findings, including gangrenous necrosis, chronic osteomyelitis, calcific atherosclerosis, and tibial vessel occlusions. This case showcases how blue toe syndrome can progress despite source control when severe distal runoff disease, poor wound oxygenation, and recurrent infections are present.

## Introduction

Blue toe syndrome refers to the sudden onset of painful blue discoloration of the toes in the absence of major trauma or cold injury [1]. It is a clinical presentation that may result from impaired blood flow to the digital arteries due to conditions such as cholesterol crystal embolization, vasculitis, or other vessel-occlusive processes [1,2]. In atheroembolic disease, material from an ulcerated atherosclerotic plaque can travel from a source such as the aorta or iliac artery into smaller vessels, which can produce ischemia in the toes and forefoot area [2,3].

One characteristic feature of blue toe syndrome is the apparent preservation of proximal large-vessel circulation despite evidence of distal digital ischemia. Patients may have palpable proximal pulses, and arterial duplex studies may not show major arterial occlusion despite clinically evident digital ischemia [1,2]. Distal embolization can affect smaller vessels supplying the toes while proximal arteries remain patent, allowing pulses to remain palpable despite digital ischemia. This can make diagnosis difficult since the clinical appearance is more severe than large-vessel imaging suggests. Evaluation therefore often needs attention to both the suspected proximal embolic source and the quality of the distal runoff to the foot.

The clinical course of blue toe syndrome can range from local digital ischemia to progressive tissue loss. When the digital perfusion is poor, patients may develop nonhealing wounds, gangrene, infection, and osteomyelitis. The condition can overlap clinically with peripheral arterial disease that is associated with gangrene, rest pain, impaired healing, and increased risk of amputation [4]. Successful management of blue toe syndrome therefore involves treating the embolic source while also considering whether the distal circulation and tissue oxygenation are sufficient for wound healing. Although treatment of the proximal embolic source may reduce further embolization, restoration of distal tissue perfusion is not guaranteed when significant distal arterial disease or microvascular compromise persists.

We present a case of atheroembolic blue toe syndrome in a 72-year-old woman with peripheral arterial disease, diabetes mellitus, antiphospholipid syndrome, and prior thromboembolic disease. Despite endovascular aortic source control and multiple limb salvage attempts, the course was complicated by severe distal runoff disease, poor oxygenation, infection, osteomyelitis, and definitive below-knee amputation. This case highlights how blue toe syndrome may progress to limb loss when proximal source control is achieved, but distal perfusion and wound-healing conditions are unfavorable.

## Case presentation

A 72-year-old woman with a history of peripheral arterial disease, diabetes mellitus, antiphospholipid syndrome, and prior thromboembolic disease presented on day 0 with painful blue discoloration of the left third toe. The initial lower extremity arterial duplex ultrasound showed no significant stenosis or arterial occlusion, although there was clear ischemic discoloration. Computed tomography angiography (CTA) of the abdomen, pelvis, and bilateral extremity runoff performed on day 2 showed distal aortic mural soft plaque, with distal tibial and pedal arterial disease. These findings were consistent with atheroembolic blue toe syndrome, with the aortic lesion considered a suspected proximal embolic source in the setting of distal tibial and pedal arterial disease. Clinical photographs demonstrate the progression from initial digital discoloration to a nonhealing wound and eventually the postoperative below-the-knee amputation outcome (Figure 1).

**Figure 1:**
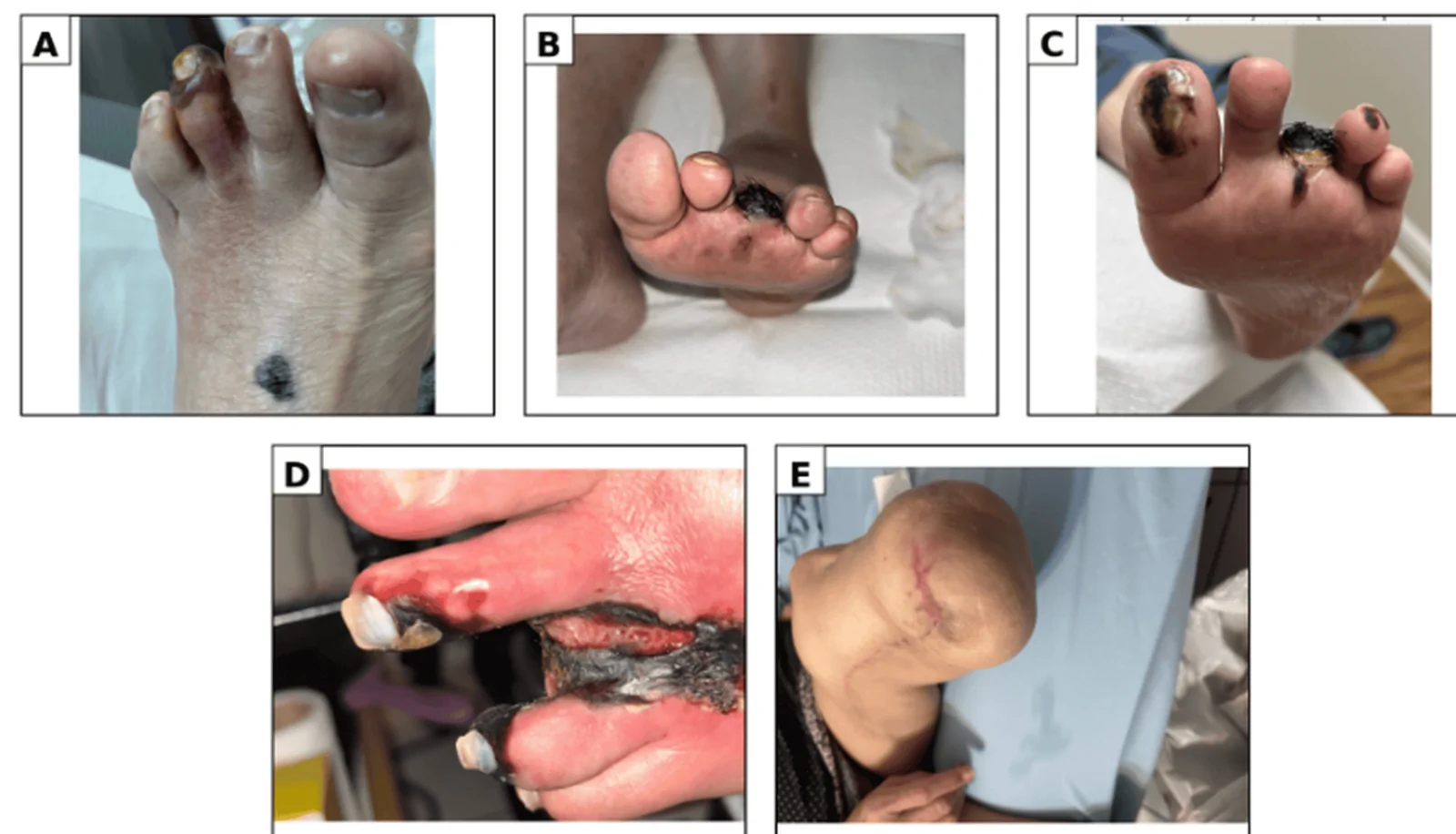
Clinical progression of left foot ischemia These clinical photographs demonstrate progression from initial dusky discoloration of the left third toe to worsening forefoot gangrene and postoperative below-knee amputation outcome. (A) Initial dusky-blue discoloration and ischemic change of the left third toe. (B) Nonhealing post-amputation wound at the left third toe region. (C) Progressive ischemic changes with additional digital gangrene and tissue loss. (D) Advanced forefoot tissue necrosis and gangrenous wound progression. (E) Postoperative appearance of the left below-knee amputation stump.

The patient underwent endovascular aortic repair on day 22 for proximal source control of the aortic lesion. A follow-up CTA on day 37 showed a patent infrarenal aortic endovascular graft with bilateral vessel runoff, although there was mild to moderate stenosis of the left dorsalis artery. Even with source control, the left third toe progressed to dry gangrene and required partial third toe amputation and debridement on day 38. Pathology from the partial amputation showed dry gangrene with necrotic bone, skin, and soft tissue, along with ulceration, necrosis, suppuration, and congestion. CTA imaging demonstrated the suspected infrarenal aortic lesion and subsequent endovascular source control (Figure 2).

**Figure 2:**
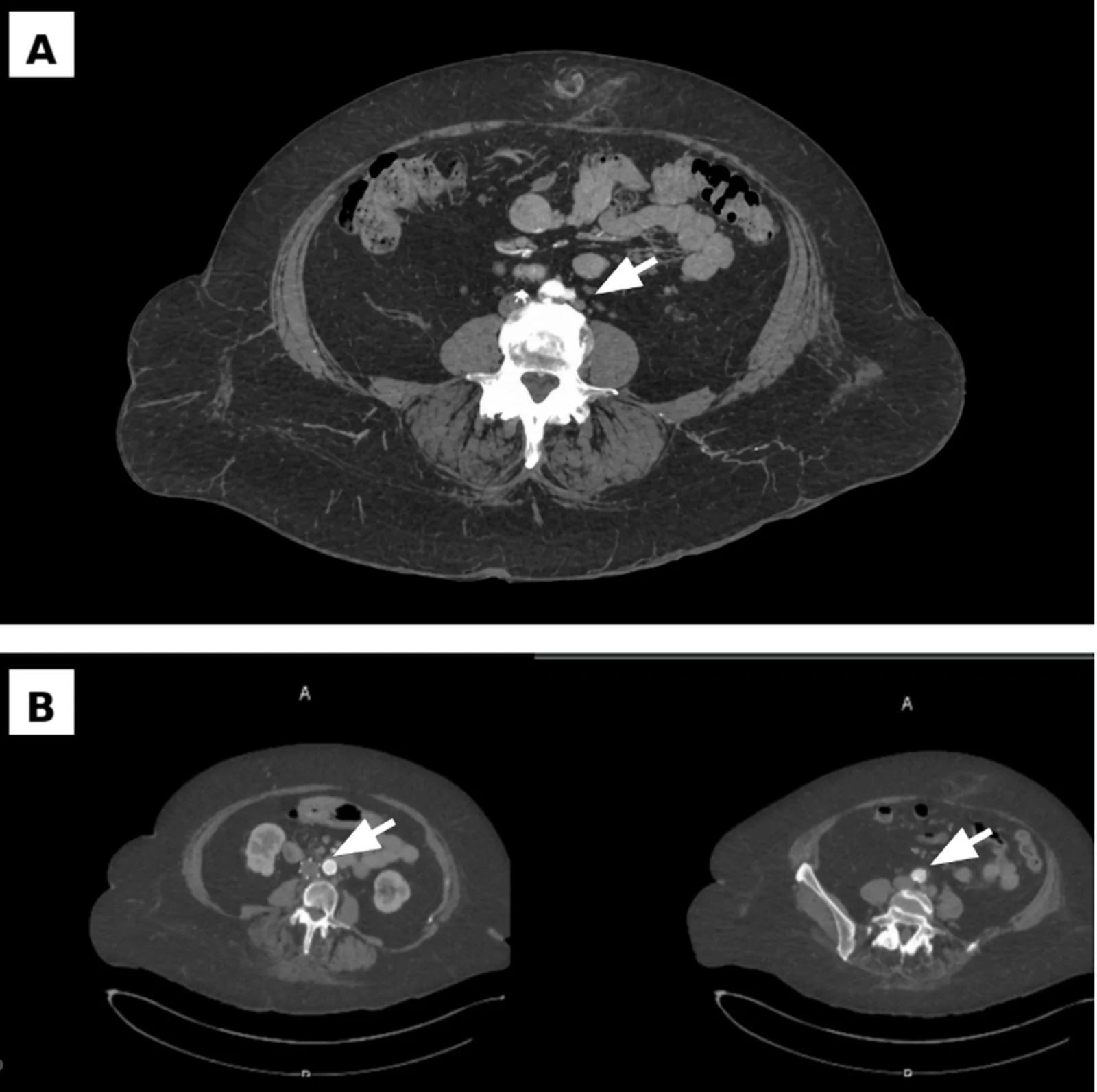
CTA evidence of an aortic embolic source and subsequent endovascular source control (A) Initial CTA (day 2) demonstrating an infrarenal aortic mural thrombus/soft plaque (arrow). (B) Follow-up CTA (day 37) demonstrating successful endovascular treatment of the infrarenal aortic lesion, with the treated aortic segment indicated (arrows). CTA: computed tomography angiography

Following the initial episode of blue toe syndrome, the amputation site failed to heal, with progressive tissue loss and concern for inadequate distal perfusion. On day 149, she underwent an additional left third-toe amputation for progressive gangrene, and pathology from this procedure showed focal osteomyelitis involving bone and cartilage. This represented progression from ischemic tissue loss to a chronic nonhealing wound now complicated with bone infection.

Beginning on day 299, the patient developed recurrent left foot ulceration and infection. The arterial duplex showed abnormal distal arterial waveforms without complete arterial occlusion. Deep wound culture grew moderate *Pseudomonas aeruginosa* and rare methicillin-resistant *Staphylococcus aureus* (MRSA), with no anaerobes isolated. She underwent aortography, left lower extremity angiography, anterior tibial/dorsalis pedis angioplasty, and tibial angioplasty on day 302 as a limb salvage revascularization attempt. She underwent debridement of tissues with partial left third metatarsal resection on day 305.

During a readmission to the hospital, the patient had worsening left foot pain and ulceration. Further imaging demonstrated postsurgical changes with soft-tissue ulceration and vascular calcifications. Superficial wound cultures grew moderate *Pseudomonas aeruginosa*. Bilateral lower-extremity arterial duplex on day 321 showed hemodynamically significant stenosis between the left proximal and distal popliteal arteries, with post-stenotic monophasic waveforms and decreased infrapopliteal velocities. Wound-care evaluation later that month documented worsening ischemic changes of the distal forefoot, wound dehiscence, dry gangrene of additional digits, and mottling of the fifth digit. Figure 3 compares early post-amputation findings with later changes in the left foot.

**Figure 3:**
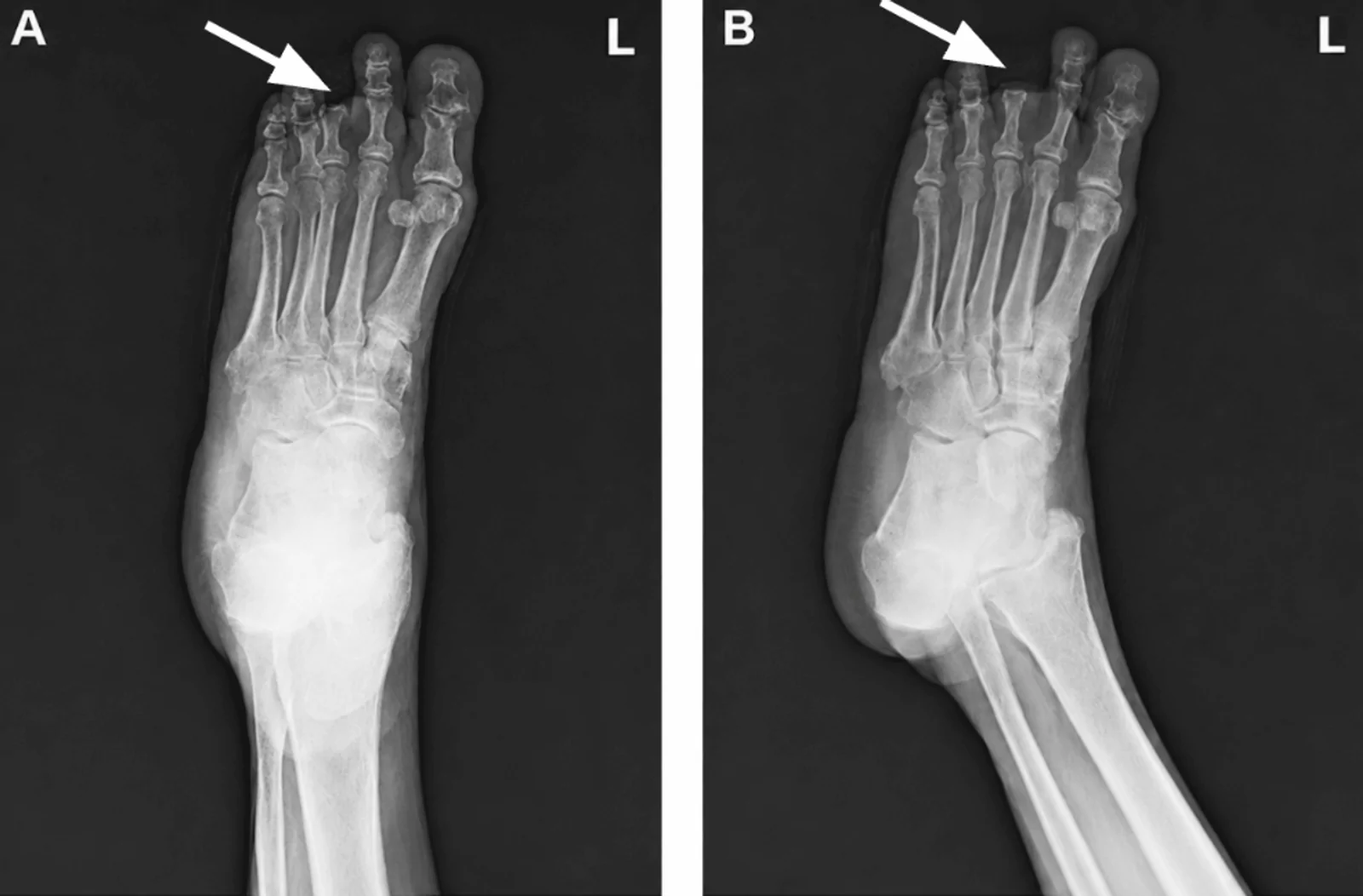
Comparison of left foot radiographs following amputation (A) Early post-amputation radiograph (day 39) demonstrating the third ray amputation site (arrow). (B) Follow-up radiograph (day 321) of the corresponding third ray region (arrow) for interval comparison.

The patient was admitted to a new hospital on day 345 with progressive pain, erythema, swelling, gangrene, soft-tissue infection, and peripheral arterial disease. There was also concern for osteomyelitis. Perfusion testing showed severe distal disease. Ankle-brachial index (ABI) and pulse volume recording (PVR) testing on day 346 demonstrated a left ABI of 0.50, a left metatarsal/dorsalis pedis index of 0.64, severely decreased left metatarsal PVR, and a non-pulsatile left great toe waveform. Angiography demonstrated patent vessels to the knee but severe distal runoff compromise, including occluded tibial vessels and diminutive plantar/dorsalis pedis reconstitution. Transcutaneous oxygen measurement (TCOM) testing demonstrated poor local oxygenation, including profound hypoxia at the left distal medial dorsal foot. Deep wound culture grew significant *Pseudomonas aeruginosa*. Magnetic resonance imaging (MRI) showed multifocal devitalized tissue/dry gangrene with acute osteomyelitis of multiple toes.

Given the severity of distal ischemia and infection, aggressive limb-salvage procedures were attempted. On day 350, the patient underwent amputation of toes 1-5, common plantar embolectomy, and a below-knee popliteal-to-common plantar bypass. On day 356, she received a transmetatarsal amputation, Achilles tendon lengthening, external fixation, and antibiotic bead placement. Pathology from the transmetatarsal amputation demonstrated acute osteomyelitis, focal soft-tissue necrosis, acute and chronic inflammation, and a proximal bone margin negative for osteomyelitis.

Despite these interventions, the transmetatarsal amputation site developed a nonhealing infection and progressive ischemia. On day 367, the patient underwent a definitive left transtibial/below-knee amputation. Operative findings included purulent drainage from the lateral transmetatarsal amputation wound. A clotted graft without blood flow and a calcified, nonbleeding posterior tibial vessel were also identified. At the transtibial amputation level, the tissue was viable, and no infection was identified. Final pathology demonstrated gangrenous necrosis, chronic osteomyelitis, calcific atherosclerosis, tibial vessel calcific occlusions, and viable proximal skin and soft-tissue margins. The overall clinical course is summarized in Table 1, while key diagnostic findings demonstrating persistent distal ischemia, impaired wound healing, infection, and osteomyelitis are summarized in Table 2.

**Table 1:** Chronological summary of clinical course CTA: computed tomography angiography; TCOM: transcutaneous oxygen measurement; TMA: transmetatarsal amputation

Clinical event	Key findings	Clinical significance
Days 0-2: initial presentation and vascular workup	The patient presented with a dusky-blue left third toe. Duplex showed no major arterial occlusion; CTA demonstrated infrarenal aortic mural thrombus/soft plaque with distal tibial/pedal disease	Blue toe syndrome with suspected aortic embolic source and distal runoff disease
Day 22: endovascular aortic repair	Endovascular repair of infrarenal aortic ulceration with associated thrombus	Proximal embolic source treated
Days 36-151: progressive tissue loss and toe amputation	The third toe progressed to gangrene despite treatment of the proximal aortic source, requiring partial amputation. The amputation wound failed to heal and subsequently required further amputation; pathology demonstrated focal osteomyelitis	Progressive distal tissue loss despite source control
Days 285-345: recurrent ischemic/infected wound	Abnormal distal arterial flow, recurrent infection, distal angioplasty, debridement, and partial third-metatarsal resection	Continued wound failure and progressive tissue loss
Days 345-349: advanced ischemia and osteomyelitis	Severe distal runoff compromise, poor TCOM oxygenation, devitalized tissue, and multifocal acute osteomyelitis	Poor healing potential with advanced tissue and bone infection
Day 350: limb-salvage revascularization and toe amputations	Toes 1-5 amputated with common plantar embolectomy and popliteal-to-plantar bypass	Further limb-salvage attempt
Day 356: TMA	TMA with Achilles tendon lengthening, external fixation, and antibiotic beads; acute osteomyelitis with negative proximal margin	Partial-foot salvage attempt failed to achieve durable healing
Day 367: below-knee amputation	Nonhealing infected TMA site with pus, clotted graft, and calcified/nonbleeding posterior tibial vessel	Definitive amputation after failure of repeated limb-salvage attempts

**Table 2:** Key diagnostic findings supporting persistent distal ischemia, impaired wound healing, infection, and osteomyelitis ABI: ankle-brachial index; DP: dorsalis pedis; MRSA: methicillin-resistant *Staphylococcus aureus*; PVR: pulse volume recording; TCOM: transcutaneous oxygen measurement; BKA: below-knee amputation

Clinical event	Key findings	Clinical significance
Day 0	No arterial occlusion or hemodynamically significant stenosis despite painful dusky-blue discoloration of the left third toe	Supported blue toe syndrome despite the absence of a major proximal arterial occlusion
Day 2	Distal infrarenal aortic mural thrombus/soft plaque measuring 0.8 × 1.2 × 1.2 cm, with distal tibial/pedal disease and loss of distal left DP opacification	Identified suspected proximal embolic source with distal runoff disease
Day 37	Patent infrarenal aortic graft with bilateral three-vessel runoff; mild-to-moderate left dorsalis artery stenosis	Demonstrated proximal aortic source control with persistent distal pedal arterial disease
Day 38, reported day 43	Dry gangrene of the left third toe with alternating viable and necrotic bone, skin, and soft tissue; ulceration, necrosis, suppuration, and congestion	Confirmed tissue necrosis and dry gangrene
Day 149, reported day 151	Bone/cartilage with focal osteomyelitis; resection margin free of inflammation	Confirmed progression from dry gangrene/necrosis to bone infection
Day 299	Moderate *Pseudomonas aeruginosa* and rare MRSA; no anaerobes isolated	Confirmed infection of the nonhealing ischemic wound
Day 346	Left ABI 0.50; left metatarsal/DP index 0.64; severely decreased left metatarsal PVR; non-pulsatile left great toe waveform	Demonstrated severe distal perfusion impairment before final limb-salvage attempts
Day 347	Patent vessels to the knee with severe distal runoff compromise, including occluded tibial vessels and diminutive plantar/DP reconstitution	Demonstrated severe distal/pedal arterial disease limiting wound healing and limb salvage
Day 348	Profound TCOM hypoxia of the left foot; heavy *Pseudomonas aeruginosa* growth	Confirmed poor wound-healing potential and heavy gram-negative infection during the final admission
Day 356, reported day 361	Acute osteomyelitis with focal soft-tissue necrosis and acute/chronic inflammation	Confirmed acute osteomyelitis and ongoing tissue necrosis despite attempted limb salvage
Day 367, reported day 375	Gangrenous necrosis, chronic osteomyelitis, calcific atherosclerosis, viable proximal skin/soft-tissue margins, and calcific occlusions up to 50% in anterior tibial vessels and up to 70% in posterior tibial vessels	Final evidence of persistent distal disease; viable proximal margins supported tissue viability at the BKA level

## Discussion

Aortic atheroembolism is an important cause of blue toe syndrome, resulting from distal embolization of cholesterol crystals from atherosclerotic plaques [5]. Although treatment of the proximal embolic source may prevent further embolization, established distal tissue injury may persist despite successful source control. A similar clinical course has been reported in a patient with cholesterol crystal embolization and chronic limb-threatening ischemia who experienced recurrent gangrene, osteomyelitis, and eventual amputation despite repeated endovascular treatment [6]. CTA identified an infrarenal aortic mural thrombus/soft plaque with concomitant distal tibial and pedal disease, and endovascular repair was subsequently performed to address the suspected proximal embolic source. Despite treatment of the proximal lesion, the patient developed progressive digital gangrene and a nonhealing amputation wound. This course highlights the potential disconnect between treatment of a suspected proximal embolic source and recovery of already compromised distal tissue.

Objective assessment of post-procedural tissue perfusion may provide important prognostic information regarding wound healing in patients with chronic limb-threatening ischemia, as revascularization does not guarantee healing and outcomes depend on multiple local and systemic factors [7]. In one series using skin perfusion pressure (SPP), early wound healing was achieved in 84.4% of limbs with a post-procedural SPP of 50 mmHg or higher, compared with 35.0% of limbs with values below that threshold [8]. Although SPP was not used, these findings support the broader importance of assessing tissue-level perfusion after revascularization. In this patient, subsequent vascular evaluation demonstrated an ABI of 0.50, severe distal runoff compromise on angiography, and markedly impaired tissue oxygenation on TCOM, providing objective evidence of persistent distal perfusion abnormalities that may have contributed to the patient's progressive tissue loss and failure to heal.

The persistent perfusion deficit also left the wound vulnerable to infection. Inadequate tissue oxygenation can impair wound healing and host defense, making ischemic wounds more susceptible to persistent infection [9]. In this patient, recurrent wound infection, including *Pseudomonas aeruginosa* and MRSA, developed during the course of progressive tissue loss. Subsequent pathology demonstrated osteomyelitis. Antimicrobial-resistant and polymicrobial infections have also been associated with delayed wound healing after minor amputation in chronic limb-threatening ischemia [10]. The persistence of infection alongside severe distal perfusion impairment was temporally associated with continued wound failure despite repeated debridement and revascularization attempts.

In this setting of ongoing distal ischemia and infection, repeated attempts at limb salvage were ultimately unsuccessful. Revascularization can improve perfusion and facilitate wound healing in chronic limb-threatening ischemia; however, the likelihood of successful limb salvage depends on factors including distal arterial runoff, tissue perfusion, infection, and the extent of tissue loss. Increasing severity of ischemia and foot infection has been associated with higher rates of major amputation and longer wound-healing times [11]. In this patient, treatment progressed from distal angioplasty and plantar embolectomy to popliteal-to-plantar bypass, staged toe amputations, and transmetatarsal amputation with antibiotic bead placement. Despite these interventions, the patient developed persistent ischemia, recurrent infection, osteomyelitis, and nonhealing tissue, ultimately requiring below-knee amputation. The repeated failure of limb-salvage procedures illustrates the difficulty of achieving healing in the setting of severe distal arterial disease and established tissue injury.

This case illustrates that treatment of the suspected embolic source does not necessarily result in recovery of already ischemic distal tissue. Despite successful aortic repair, the patient continued to develop distal ischemia, recurrent infection, and osteomyelitis and ultimately required multiple limb-salvage procedures followed by below-knee amputation. Recognizing this distinction between proximal source control and distal tissue viability may help clinicians better assess wound-healing potential and determine the feasibility of limb salvage in similar cases.

## Conclusions

This case has demonstrated that blue toe syndrome can progress to major limb loss despite various interventions and treatment of the suspected embolic source. Although endovascular aortic repair addressed the suspected proximal embolic source, the patient's condition continued to worsen with progressive distal ischemia, recurrent infection, osteomyelitis, and nonhealing tissue loss. The case emphasizes that proximal source control does not guarantee wound healing when distal runoff disease and tissue-level perfusion remain poor.

In patients with blue toe syndrome and progressive tissue loss, healthcare professionals should evaluate not only the embolic source but also distal perfusion, wound oxygenation, infection burden, and the feasibility of viable limb salvage. In this case, recognition of severe distal perfusion impairment, poor tissue oxygenation, and ongoing infection provided clinically relevant information regarding the limited potential for successful wound healing during continued limb-salvage efforts. Below-knee amputation ultimately provided definitive control after multiple unsuccessful foot-salvage procedures.
